# Feasibility and Compatibility of Minilaparotomy Hysterectomy in a Low-Resource Setting

**DOI:** 10.1155/2018/8354272

**Published:** 2018-08-01

**Authors:** Abhilasha Agarwal, Jyothi Shetty, Deeksha Pandey, Gazal Jain

**Affiliations:** Kasturba Medical College, Manipal Academy of Higher Education, Manipal, India

## Abstract

**Introduction:**

Minilaparotomy hysterectomy (MLH) relies on the simplicity of the traditional open technique of abdominal hysterectomy, imparts cosmesis and faster recovery of laparoscopic hysterectomy yet avoids the long learning curve and cost of expensive setup and instrumentation associated with the minimally invasive approaches, namely, laparoscopy and robotics. In the present study, we tried to ascertain whether the results obtained with MLH can be compared to LAVH in terms of its feasibility, intraoperative variables, and complications. The null hypothesis was that both MLH and LAVH are comparable techniques; thus, where cost and surgeon's experience are the confining issues, patients can be reassured that MLH gives comparable results.

**Materials and Methods:**

This was a prospective observational study done over a period of two years at a university teaching hospital. A total of 65 patients were recruited, but only 52 (MLH: 27; LAVH: 25) could be included in final analysis. All surgeries were performed by one of the two gynecologists with almost equal surgical competence, and outcomes were compared.

**Results:**

MLH is a feasible option for benign gynecological pathologies as none of the patients required increase in the initial incision (4–6 cm). MLH could be done for larger uteri (MLH: 501.30 ± 327.96 g versus LAVH: 216.60 ± 160.01 g; *p* < 0.001), in shorter duration (MLH: 115.00 ± 21.43 min versus LAVH 172.00 ± 27.91 min; *p* < 0.001), with comparable blood loss (MLH: 354.63 ±227.96 ml; LAVH: 402.40 ± 224.02 ml; *p*=0.334), without serious complications when compared to LAVH.

**Conclusion:**

The technique of MLH should be mastered and encouraged to be used in low-resource setting to get results comparable to laparoscopic surgery. This trial is registered with NCT03548831.

## 1. Introduction

Hysterectomy is the second most frequently performed major surgical procedures on women all over the world, next only to cesarean. However, there is no consensus regarding the route and/or the technique of hysterectomy [1]. Enthusiastic surgeons and inquisitive investigators are still busy trying to find out the most optimal way to perform a hysterectomy. While classical or conventional abdominal hysterectomy is easy to perform and easy to learn, the laparoscopic one provides benefits of better cosmesis and shorter hospital stay. Recently minilaparotomy hysterectomy (MLH) has come up as a middle path coupling cosmesis and faster recovery with less dependence on technology and instrumentation [1–3]. This approach relies on the simplicity of the traditional open technique of abdominal hysterectomy and avoids the long learning curve and cost of expensive setup and instrumentation associated with laparoscopic hysterectomy [3].

In the Cochrane review, though vaginal approach has been suggested as the best approach for performing a hysterectomy for benign gynecological conditions, it is not always feasible to adopt this route. The second best option is the laparoscopic route; however, total laparoscopic hysterectomy (TLH) is associated with more urinary tract injuries [4]. Laparoscopic-assisted vaginal hysterectomy (LAVH) is the combination of these two wherein up to skeletonization of uterine arteries is performed laparoscopically and the rest of the hysterectomy is completed vaginally. This approach reduces the risk of ureteric injuries, as seen in TLH. However, still, the setup, cost, duration of surgery, and surgical experience are the confounding factors to make it a universal standard of care.

In the present study, we tried to ascertain whether the results obtained with MLH can be compared to LAVH in terms of feasibility, intraoperative variables (like duration of surgery and amount of blood loss), and complications. The null hypothesis is that both MLH and LAVH are comparable techniques; thus, where cost and surgeon's experience are the confining issues, patients can be reassured that MLH gives comparable results.

## 2. Materials and Methods

This was a prospective observational study performed at a university teaching hospital, from August 2014 to July 2016. Approval from Institutional Ethical Committee was obtained (IEC 429/2014). Patients who required hysterectomy for benign gynecological conditions (with uterine size up to 20 weeks) but had no uterine descent on examination were recruited. All these patients were informed in detail about the variously available modalities of hysterectomy, with their pros and cons as per the available literature. The final decision was made on a joint consensus between the patient and the surgeon keeping the cost, pathology (e.g., endometriosis encouraged for LAVH), and comorbidities (e.g., cardiorespiratory conditions contradicting pneumoperitoneum encouraged for MLH) in mind. Informed written consent was obtained from all patients.

### 2.1. Intervention

All patients followed the same standard preoperative protocol. All surgeries were performed under general anesthesia with endotracheal intubation. Demographic details that included age, parity, body mass index (BMI), baseline investigations, diagnosis, and comorbidities were collected a day prior to the day of surgery. Based on our initial experience with MLH to reduce intraoperative blood loss, in cases of larger uteri wherein need to debulking prior to clamping of uterine arteries was anticipated; around one hour prior to surgery, 1 g tranexamic acid in 100 ml of saline was administered intravenously and 400 mcg of misoprostol was inserted vaginally. Intraoperative variables (duration of surgery—initiation of skin incision to finishing skin suturing, blood loss, uterine weight, visceral injuries, and conversion rate) were noted down in accordance with the anesthesiologist to reduce the operator's bias. All surgeries were performed by two gynecologists (JS and DP) with an almost equal level of surgical competence. Postoperative pain, need of analgesia, complications, and duration of hospital stay were also recorded.

### 2.2. Surgical Technique

The details of the surgical technique followed are described in the following sections.

### 2.2.1. MLH

**Figure 1:**
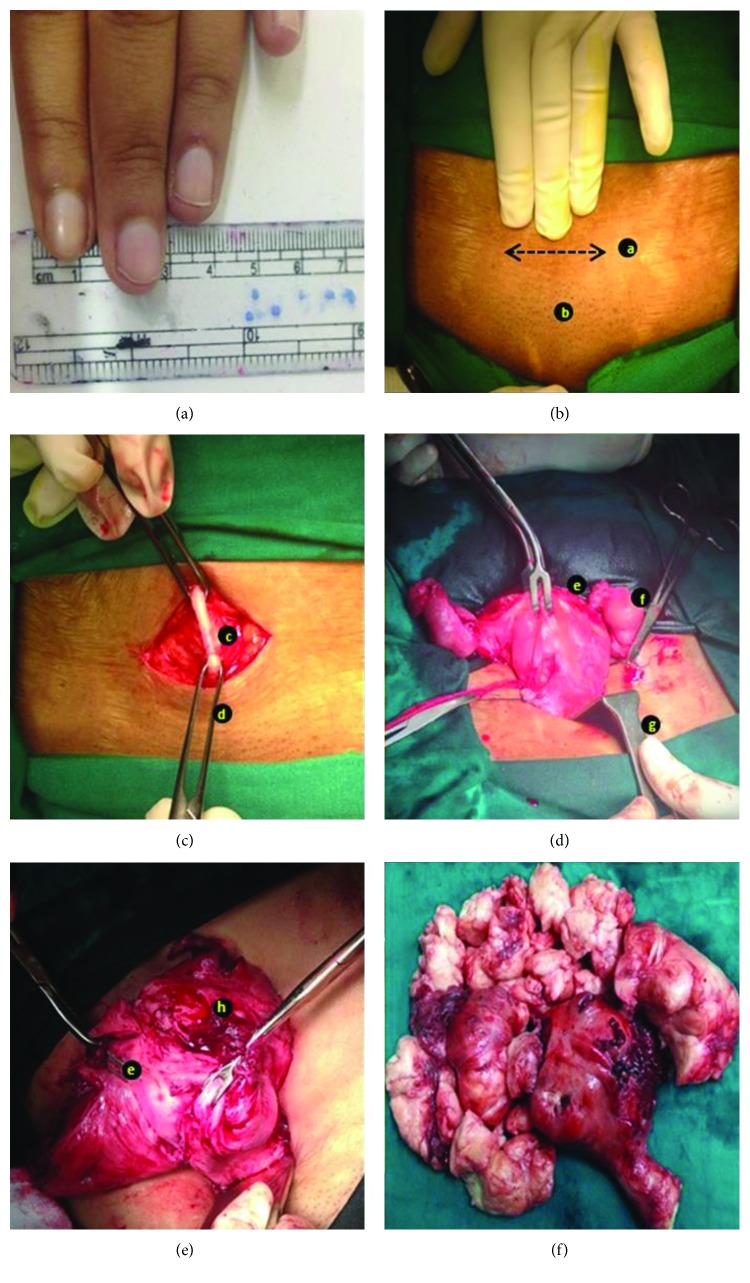
Pictorial representation of 2 representative cases of MLH describing important steps of our technique. (a) Approximation of size. (b) Deciding the abdominal incision: a, 4 cms abdominal incision; b, pubic symphysis. (c) Opening the abdomen in layers: c, rectus sheath; d, allis forceps. (d) Stepwise clamping in uterus size up to 12 weeks: e, bulldog clamp for traction; f, hydrosaplinx; g, right-angled retractor. (e) Volume reduction prior to the clamping the pedicles for hysterectomy—in a large uterus: h, debulking procedure. (f) Final retrieved specimen of one of the large uteri (weight: 1.3 kg).

The surgery was performed in the low lithotomy position, to facilitate vaginal uterine manipulation if required. A transverse incision of 4–6 cm was made, 2 cm above the pubic symphysis. Underlying rectus sheath was incised transversely 6–8 cm (1 cm more on either side as compared to the skin incision). After opening the peritoneum vertically, abdominal wall was retracted using thin Deaver and/or Richardson retractors. Fundus of the uterus or anterior uterine wall (where fundus was high up, in cases of large uteri) was held with a bulldog clamp in order to facilitate mobilization. In cases where the uterus was up to 12 weeks' size, it was possible to hold the fundus and sequentially clamp the ligaments till uterine arteries as per the standard protocol, at least on one side. After which, the uterus was pulled out from the detached side and successive clamping was done on the other side. Where the uterus was bigger and it was not possible to reach its fundus, debulking was done by performing myomectomy (in cases of multiple fibroids) or by the helical incision technique [5]. After optimal debulking when the uterus could be exteriorized, hysterectomy was performed as per the standard protocol (Figure 1).

### 2.2.2. LAVH

In LAVH with the help of a 10 mm principal trocar and three ancillary 5 mm trocars, the procedure was performed up to the skeletonization of uterine vessels. The remaining part was completed vaginally. If required, volume reduction procedures like bisection, myomectomy, or coring were performed for specimen retrieval through the vagina.

### 2.3. Statistical Analysis

Statistical Package for Social Sciences (SPSS 20 for windows) was used for data compilation and statistical analysis. The independent sample *t*-test was used for continuous variables like age, BMI, and operative time and to test the difference between mean values of the variables in the two groups compared. This was also applied for the comparison of pain obtained by the Visual Analogue Scale (VAS), between the groups. The Mann–Whitney test was used to analyze variables that had a nonparametric distribution that included intraoperative blood loss and weight of the specimen.

## 3. Results

**Figure 2:**
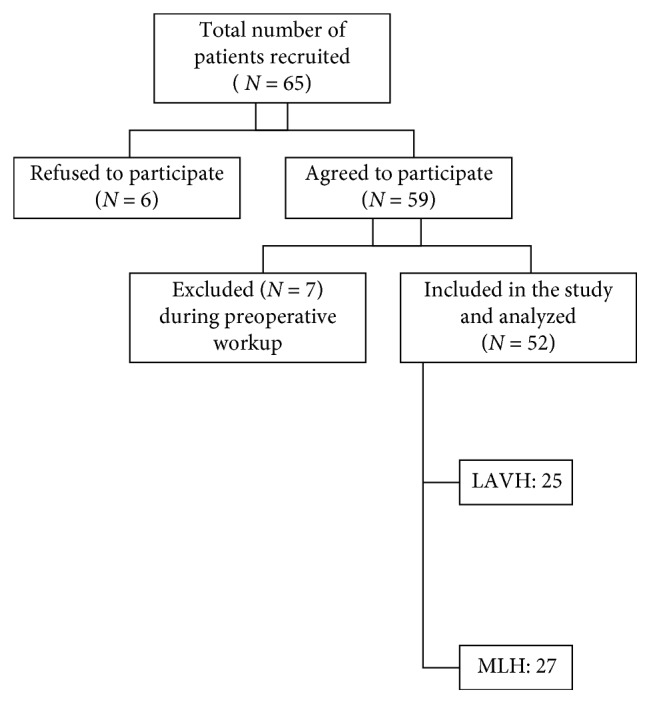
Recruitment and patient allotment through the study.

A total of 65 women were recruited for the study who were admitted to undergo hysterectomy for benign gynecological pathology and fulfilled the inclusion criteria. After thorough information about the study, six women refused to participate, while 59 agreed. Among these 59 women, during the preoperative workup, seven other patients had to be excluded. One was found to have cholelithiasis incidentally and decided to undergo cholecystectomy in the same sitting, and one more patient decided to have the umbilical hernia repair. Two women were found to be having previously undiagnosed hypothyroidism—thus they were started on medication and the surgery was postponed. One patient was detected to have retropositive status. Two other patients were not found to be fit for general anesthesia during their preanesthetic checkup, and for them, the plan was changed and hysterectomy was performed under spinal anesthesia. Thus, a total of 52 patients were finally included in the study, wherein 27 underwent MLH and 25 had LAVH (Figure 2).

Mean age of women in the two groups (MLH: 44.75 ± 5.03 years; LAVH: 47.20 ± 6.26 years; *p*=0.123) was comparable. Around two-thirds of women in both the groups were parous (70.3% in MLH group and 76% in LAVH group). Mean BMI was also statistically comparable in the two groups (MLH: 24.84 ± 3.63 kg/m^2^; LAVH: 27.61 ± 4.90 kg/m^2^; *p*=0.024). However, if we notice the range in the upper limit of BMI, in the LAVH group, it was higher (37.8 kg/m^2^) as compared to the upper limit in the MLH group (31.5 kg/m^2^). This may be due to the selection bias. Around 20% of women in both the groups had the history of previous abdominopelvic surgeries (Table 1).

The most common indication was fibroid uterus in both the groups. However, while in the MLH group, 92.6% (*n*=25) women underwent hysterectomy for fibroid, in the LAVH group, only 36% (*n*=9) had fibroid uterus. This again points towards the selection bias of larger uteri for MLH. The other indications for hysterectomy in our cohort were adenomyosis (MLH: 2 (7.4%); LAVH: 7 (28%)), DUB refractory to medical management (LAVH: 3 (12%)), endometrial hyperplasia (2 cases both in the LAVH group: one was simple hyperplasia with atypia and another one was complex hyperplasia without atypia), high-grade cervical intraepithelial neoplasia (CIN 2 (*n*=1) and CIN 3 (*n*=1)—both in the LAVH group), and benign ovarian tumors (*n*=2 in the LAVH group).

Intraoperatively, the mean estimated blood loss was statistically comparable in the two groups (MLH: 354.63 ±227.96 ml; LAVH: 402.40 ± 224.02 ml; *p*=0.334). However, careful observation of the range reveals that the minimum blood loss in MLH was 80 ml compared to the minimum blood loss of 150 ml in LAVH. The upper limit in both the groups was same. The difference in duration of surgery from the initiation of the abdominal incision to finishing the abdominal skin suturing (port closure in case of LAVH) was found to be lesser in MLH (115.00 ± 21.43 min) as compared to LAVH (172.00 ± 27.91 min). The range varied from 60 to 150 min in MLH and from 120 to 200 min in LAVH. This suggests that the minimum time taken in LAVH was still double the minimum time taken to complete MLH. The weight of the retrieved specimen following surgery was more in the MLH group, and the difference was statistically significant (MLH: 501.30 ± 327.96 g versus LAVH: 216.60 ± 160.01 g; *p* < 0.001). Noticeably, the largest uterus removed in the MLH group (1300 g) was around double the maximum size retrieved by LAVH (850 g). (Table 2).

During the surgery, there was no visceral injury in either of the groups. In the LAVH group, two cases had to be converted to laparotomy—one because of uncontrolled hemorrhage and the other because of dense adhesions. In none of the MLH case, there was a need to increase the initial incision; thus, there was no conversion (Table 3).

**Figure 3:**
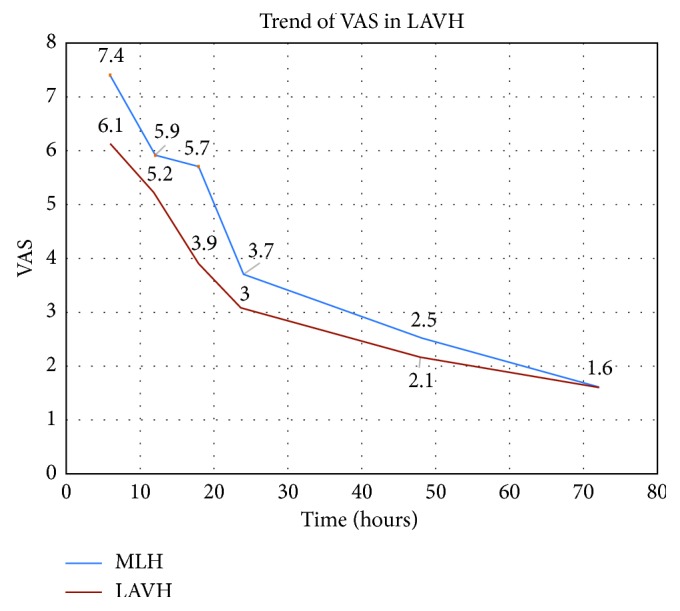
Trend of pain perceived in the two groups, as per the Visual Analogue Scale (VAS).

Postoperative pain perception was measured by VAS. As shown in Figure 3, pain perceived (represented as mean ± SD on VAS) on the immediate postoperative period, the first and second postoperative days, was significantly lower in the LAVH group. On the third postoperative day, however, the pain scores were same in both the groups.

In the postoperative period in the MLH group, one patient had UTI requiring antibiotics and one patient in the LAVH group had urinary retention on day 1 requiring catheterization for 24 hours. Two patients (7.4%) had surgical site infection (SSI) in the MLH group which was conservatively treated with antibiotics and daily dressing. None of them required secondary suturing, but one required prolonged hospitalization (11 days) for the same. No port site infection (SSI) was encountered in the LAVH group. Though two patients (8%) in LAVH had secondary hemorrhage (vault bleeding) and one had febrile morbidity, these cases also were managed conservatively with antibiotics and/or hemostatic agents, and these minor issues did not necessitate prolonged hospital stay.

Mean duration of hospital stay was 5.7 ± 1.95 days in MLH and 5.68 ± 2.17 in LAVH. Two patients in the MLH group required prolonged stay—one for management of preexisting systemic lupus erythematosus (SLE) and one because of SSI (12 days and 11 days, resp.). In the LAVH group, three patients had to stay for a longer period. One who had a conversion to laparotomy, developed a fever on day 4 (13 days stay), one had poorly controlled diabetes (10 days), and one had a personal wish as she hailed from a far off place with inadequate medical facilities in case the need arises (9 days).

## 4. Discussion

In this study, we found that MLH is a feasible option for benign gynecological pathologies, which can be done for larger uteri, in shorter duration, with less to comparable blood loss, without serious complications when compared to LAVH.

MLH has been a known and studied technique to perform a hysterectomy for benign gynecological pathologies since the late twentieth century [6]. In the early twenty-first century, Pelosi and Pelosi popularized their technique of hysterectomy through a small abdominal incision (3–6 cm small minilaparotomy and 7–8 cm large minilaparotomy) [3]. We used a 4–6 cm incision as described in Figure 1. Some surgeons prefer to use a soft, sleeve-type self-retaining abdominal retractor (used by colorectal surgeons) which facilitates the procedure [7]. However, in our experience, we did not use this disposable retractor but performed the procedure with the help of conventional metal retractors, changing their positions as and when required.

It is being suggested that minilaparotomy is a minimally invasive procedure ideal for gynecologists who are less skilled in vaginal or laparoscopic surgery and who are more comfortable with the (standard) abdominal approach [7]. This approach is not only used for hysterectomy but also found to be the feasible option for myomectomies too [8, 9], with a report of removal of myoma weighing 4.5 kg through the minilaparotomy technique [10].

As in our study, other studies also found that, compared with minilaparotomy, laparoscopic hysterectomy is associated with shorter length of hospital stay, longer operating time, and no increased patient morbidity including intraoperative and postoperative complications, emergency visits, readmissions, or repeat operations [1, 11, 12]. These studies also reported higher blood loss in cases of MLH as compared to laparoscopy. However, in our study, the difference was not significant even when we had larger uteri and more volume reduction procedures in the MLH group. This can be justified by the little modification in preoperative preparation we adopted in cases of larger uteri as has been mentioned in material and method section. One hour prior to surgery, we administered one gram of tranexamic acid intravenously and inserted 400 mcg of misoprostol vaginally. With our experience, we recommend the use of these techniques to reduce blood loss. With a little modification and adaptation of techniques like the meticulous use of retractors, mastery of volume reduction techniques, and judicious use of blood loss reducing measures, MLH can be an optimal technique providing comparable surgical results and patient satisfaction.

Furthermore, after the US Food and Drug Administration statement warning against electronic morcellation devices, minilaparotomy surgeries in gynecology have found a new interest either as a complete procedure or at least for specimen retrieval following laparoscopic/robotic surgeries. A study comparing the incidence of superficial wound complications as a result of these larger incisions as compared to the laparoscopy/robotic ports found no significant differences in the subcategories of wound complications, including cellulitis, seroma, hematoma, skin separation, wound infection, or postprocedure wound complication [13].

Studies conducted to compare the results of conventional abdominal hysterectomy and MLH concluded that MLH provides a minimal access and less invasive cost-effective option/alternative to the traditional abdominal approach obviating the need for any additional expensive equipment and, above all, improves upon the perioperative outcome, notwithstanding, whatsoever, on the quality of surgery [14, 15].

In our opinion, this is one of the best options in low-resource settings as well as for the gynecologist at the beginning of their career.

The only limitation of our study was that it was not a randomized control trial (RCT), so it had an obvious selection bias in few of the cases. Thus, the results must be interpreted with caution, before generalizing.

## 5. Conclusion

MLH is a feasible option for benign gynecological pathologies, which can be done for larger uteri, in shorter duration, with less to comparable blood loss, without serious complications when compared to LAVH. MLH also alleviates the need for costly instruments/setup and surgical experiences. This technique of hysterectomy should be mastered and encouraged to be used in low-resource setting to get results comparable to laparoscopic surgery.

## Figures and Tables

**Table 1:** Comparison of demographic characteristics among the two groups (MLH versus LAVH).

Demographic characters	MLH (*N*=27)	LAVH (*N*=25)	*P* value
Age (years)^*∗*^			
Mean ± SD	44.74 ± 5.03	47.20 ± 6.26	0.123
Median	43	47	
Range	36–60	37–63	

BMI (kg/m^2^)^*∗*^			
Mean ± SD	24.84 ± 3.63	27.61 ± 4.90	0.024
Median	25.00	27.00	
Range	18.90–31.50	19.8–37.8	

Parity (%)			
Nulliparous	02 (07.4)	02 (08)	0.9360
1–2	19 (70.3)	19 (76)	0.6289
≥3	06 (22.2)	04 (16)	0.5803

Previous pelvic surgery (%)	06 (22.2)	04 (16)	
Cesareans			
(i) Previous one cesarean	03 (11.1)	00	
(ii) Previous two cesareans	01 (03.7)	01 (4)	0.5745
Myomectomy	01 (03.7)	00	
Appendicectomy	01 (03.7)	03 (12)	

Associated comorbidities (%)			
Hypertension	02 (07.4)	00	
Diabetes	02 (07.4)	03 (12)	
Hypothyroidism	01 (03.7)	05 (20)	0.9212
Bronchial asthma	02 (07.4)	00	
Epilepsy	01 (03.7)	00	
SLE	01 (03.7)	00	

**Table 2:** Intraoperative variables among the two groups (MLH versus LAVH).

	MLH (*N*=27)	LAVH (*N*=25)	*P* value
Operating time (min)^*∗*^			
Mean ± SD	115.00 ± 21.43	172.00 ± 27.91	**<0.001**
Median	120.00	180.00	
Range	60–150	120–200	

Estimated blood loss (ml)^#^			
Mean ± SD	354.63 ± 227.96	402.40 ± 224.02	0.334
Median	300	300	
Range	80–1000	150–1000	

Weight of the uterus (g)^#^			
Mean ± SD	501.30 ± 327.96	216.60 ± 160.01	**<0.001**
Median	450.00	200.00	
Range	100–1300	75–850	

**Table 3:** Intraoperative and postoperative complications among the two groups (MLH versus LAVH).

Complications	MLH *N* = 27 (%)	LAVH *N* = 25 (%)
Intraoperative		
Vascular/visceral injury	Nil	Nil
Uncontrollable primary hemorrhage	Nil	01 (04)
Conversion to laparotomy	Nil	02 (08)

Postoperative complications		
UTI	01 (03.7)	Nil
SSI	02 (07.4)	Nil
Urinary retention	Nil	01 (04)
Secondary hemorrhage (vault bleeding)	Nil	02 (08)
Febrile morbidity	Nil	01 (04)
